# The scrambled story between hyaluronan and glioblastoma

**DOI:** 10.1016/j.jbc.2021.100549

**Published:** 2021-03-17

**Authors:** Matías Arturo Pibuel, Daniela Poodts, Mariángeles Díaz, Silvia Elvira Hajos, Silvina Laura Lompardía

**Affiliations:** 1 Departamento de Microbiología, Inmunología, Biotecnología y Genética, Facultad de Farmacia y Bioquímica, Instituto de Estudios de la Inmunidad Humoral (IDEHU)-CONICET, Universidad de Buenos Aires, Capital Federal, Argentina; 2 Instituto de Estudios de la Inmunidad Humoral (IDEHU)-CONICET, Universidad de Buenos Aires, Capital Federal, Argentina

**Keywords:** cancer, chemoresistance, glioblastoma, hyaluronan, invasion, migration, proliferation, temozolomide, tumor therapy, 4MU, 4-methylumbelliferone, ADAM10, disintegrin and metalloproteinase 10, ADAMTS, disintegrin and metalloproteinase with thrombospondin motifs, Akt, protein kinase B (PKB), AMPK, AMP-activated protein kinase, AP-1, activator protein-1, BBB, blood–brain barrier, BEHAN, brain-enriched hyaluronan binding, bFGFR, b-fibroblast growth factors receptor, CD133, cluster of differentiation 133, CD44, cluster of differentiation 44, CNS, central nervous system, CS, chondroitin sulfate, CSPG4/NG2, chondroitin sulfate proteoglycan 4, CXCL12, CXC chemokine ligand 12, CXCR4, CXC chemokine receptor 4, DCs, dendritic cells, ECM, extracellular matrix, EGFR, epidermal growth factor receptor, EMT, epithelial-to-mesenchymal transition, ERK, extracellular signal-regulated kinase, ERM, ezrin–radixin–moesin, FAK, focal adhesion kinase, FDA, Food and Drug Administration, GAG, glycosaminoglycans, GBM, glioblastoma, GPI, glycosylphosphatidylinositol, HA, hyaluronan, HA-LNPs, HA-coated lipid-based nanoparticles, HA-M, HA-decorated micelle, HAPLN, HA- and proteoglycan-link proteins, HAS, hyaluronan synthases, HAS2-AS1, antisense for HAS2, HMW-HA, high-molecular-weight HA, HS, heparan sulfate, HYAL, hyaluronidase, IDH, isocitrate dehydrogenase, IFN-γ, interferon-γ, IL-1α, interleukin 1α, iNOS, inducible nitric oxide synthase, LMW-HA, low-molecular-weight HA, MAPK, mitogen-activated protein kinases, MEK, mitogen-activated protein kinase kinase, MGMT, methyl guanine methyl transferase, MMP, matrix metalloproteinase, MT1-MMP, membrane type 1-matrix metalloproteinase, NF-κB, nuclear factor-κB, NSPC, neural stem and progenitor cells, oHA, oligomers of HA, PAI-1, plasminogen activator inhibitor-1, PBMCs, peripheral blood mononuclear cells, PCL, poly (ε-caprolactone), PDGFR, platelet-derived growth factor receptor, PI3K, phosphatidylinositol 3-kinase, PLK1, Polo-like kinase 1, PTEN, tumor suppressor phosphatase and tensin homolog, Ras, oncogenic rat sarcoma, RHAMM, HA-mediated motility receptor, RhoA, Rho GTPase A, ROK, repressor, open reading frame, kinase, RON, receptor originated from nantes, SIRT1, Sirtuin 1, TGFβR-1, transforming growth factor β receptor, TIMP-1, tissue inhibitor of metalloproteinases, TLR4, Toll-like receptor 4, TMEM, transmembrane Protein 2, TMZ, temozolomide, UDP-GlcNAc, uridine diphosphate N-acetylglucosamine, uPA, urokinase-type plasminogen activator, uPAR, urokinase-type plasminogen activator receptor, VEGF, vascular endothelial-derived growth factor

## Abstract

Advances in cancer biology are revealing the importance of the cancer cell microenvironment on tumorigenesis and cancer progression. Hyaluronan (HA), the main glycosaminoglycan in the extracellular matrix, has been associated with the progression of glioblastoma (GBM), the most frequent and lethal primary tumor in the central nervous system, for several decades. However, the mechanisms by which HA impacts GBM properties and processes have been difficult to elucidate. In this review, we provide a comprehensive assessment of the current knowledge on HA’s effects on GBM biology, introducing its primary receptors CD44 and RHAMM and the plethora of relevant downstream signaling pathways that can scramble efforts to directly link HA activity to biological outcomes. We consider the complexities of studying an extracellular polymer and the different strategies used to try to capture its function, including 2D and 3D *in vitro* studies, patient samples, and *in vivo* models. Given that HA affects not only migration and invasion, but also cell proliferation, adherence, and chemoresistance, we highlight the potential role of HA as a therapeutic target. Finally, we review the different existing approaches to diminish its protumor effects, such as the use of 4-methylumbelliferone, HA oligomers, and hyaluronidases and encourage further research along these lines in order to improve the survival and quality of life of GBM patients.

Glioblastoma (GBM), also known as grade IV astrocytoma by the World Health Organization classification (1), is the most frequent primary tumor of the central nervous system (CNS) in adults. It is characterized by fast growth, invasiveness, and high mortality, with a median survival of less than 15 months after diagnosis (2, 3). Currently there are few therapeutic options (2, 4, 5, 6, 7, 8), with the first line therapy being surgical resection and radiotherapy combined with cycles of temozolomide (TMZ). Unfortunately, TMZ therapy causes severe adverse effects, such as myelosuppression and hepatotoxicity, and almost 50% of patients exhibit resistance to the treatment (2, 4, 6, 7, 8, 9, 10, 11). Furthermore, high inter- and intratumor heterogeneity, individual variability, and different stages of disease at diagnosis time complicate GBM treatment (12).

Although tumor aggressiveness and resistance are often thought of as being intrinsic to malignant cells, there is a rising appreciation of the critical importance of the tumor microenvironment, including nontumor cells and the extracellular matrix (ECM), in tumorigenesis. In addition, the ECM plays a crucial role in drug penetration as well as in the modulation of the immune system, also impacting on the mechanisms of tumor evasion and invasion (13, 14, 15, 16, 17, 18). For these reasons, there is a growing interest in studying the CNS ECM and the mechanisms through which it impacts the development and progression of brain tumors, with obvious implications for the development of new therapeutic alternatives. This is a particularly intriguing area because the ECM in the brain differs notably from that of other organs. Thus, focused investigations into this ECM are likely to broaden our understanding of neurobiology as well as advance cancer treatments.

One of the primary components of ECM is hyaluronan (HA), which is present in the parenchymal ECM along with proteoglycans (such as aggrecan, versican, neurocan, and brevican without collagen), the tenascins and link proteins (19, 20), and other glycosaminoglycans (GAGs), including chondroitin sulfate, heparan sulfate, and keratan sulfate (19, 20).HA is a linear and nonsulfated GAG made of repetitive units of D-glucuronide acid and N-acetyl-D-glucosamine (21, 22, 23, 24). It serves as a backbone to which link proteins and proteoglycans can be attached, building a three-dimensional (3D) network (19, 20, 25, 26, 27, 28), and also serves as a ligand for the cell-surface glycoprotein CD44 and the receptor for HA-mediated cell motility (RHAMM). HA is involved in tissue organization, wound healing, leukocyte traffic, growth and cellular differentiation, and other functions (29, 30, 31). In the CNS, HA also participates in the correct generation, proliferation, and maturation of neural stem cell progenitors (NSCP) during brain development and repair (28, 32).

Reports published in the 1970s and 1980s established that HA has another, undesirable role: these studies demonstrated that production of GAGs—and particularly HA—by malignant glioma cells was higher than their production in normal glial cell lines (33, 34, 35, 36, 37), which was associated with a higher rate of cell proliferation (38). Later, studies partially contradicted these reports, showing that the addition of exogenous HA did not modify cell proliferation (39, 40), but several publications did confirm a correlation between the addition of HA and an enhanced rate of invasion across multiple glioma cell lines (39, 40, 41, 42, 43, 44, 45). Our recent data extended these conclusions, showing that both high-molecular-weight HA (HMW-HA; 1.5–1.8 × 10^6^ Da) and low-molecular-weight HA (LMW-HA; 1–3 × 10^5^ Da) enhance cell migration without modifying cell proliferation on the murine GBM cell line GL26 (46).

We now know that HA strongly impacts tumor development and progression, favoring cell proliferation, angiogenesis, lymphangiogenesis, chemotherapy resistance, evasion of apoptosis, and invasion of surrounding tissues (Fig. 1) (24, 47, 48, 49, 50, 51, 52, 53). Furthermore, HA accumulation in the tumor milieu is associated with poor prognosis in several types of tumors (54). Due to HA participation in tumor progression, various strategies to mitigate its effect have been suggested, such as the use of HA oligomers (oHA), treatment with hyaluronidase (HYAL), and the utilization of the HA-synthesis inhibitor 4-Methylumbelliferone (4MU), with promising results (55, 56, 57). However, establishing a full therapeutic strategy requires an improved understanding of the role of HA on GBM progression. This is complicated by both the complex biology of this tumor and the variety of HA effects and the redundant pathways in which this GAG is involved.

**Figure 1:**
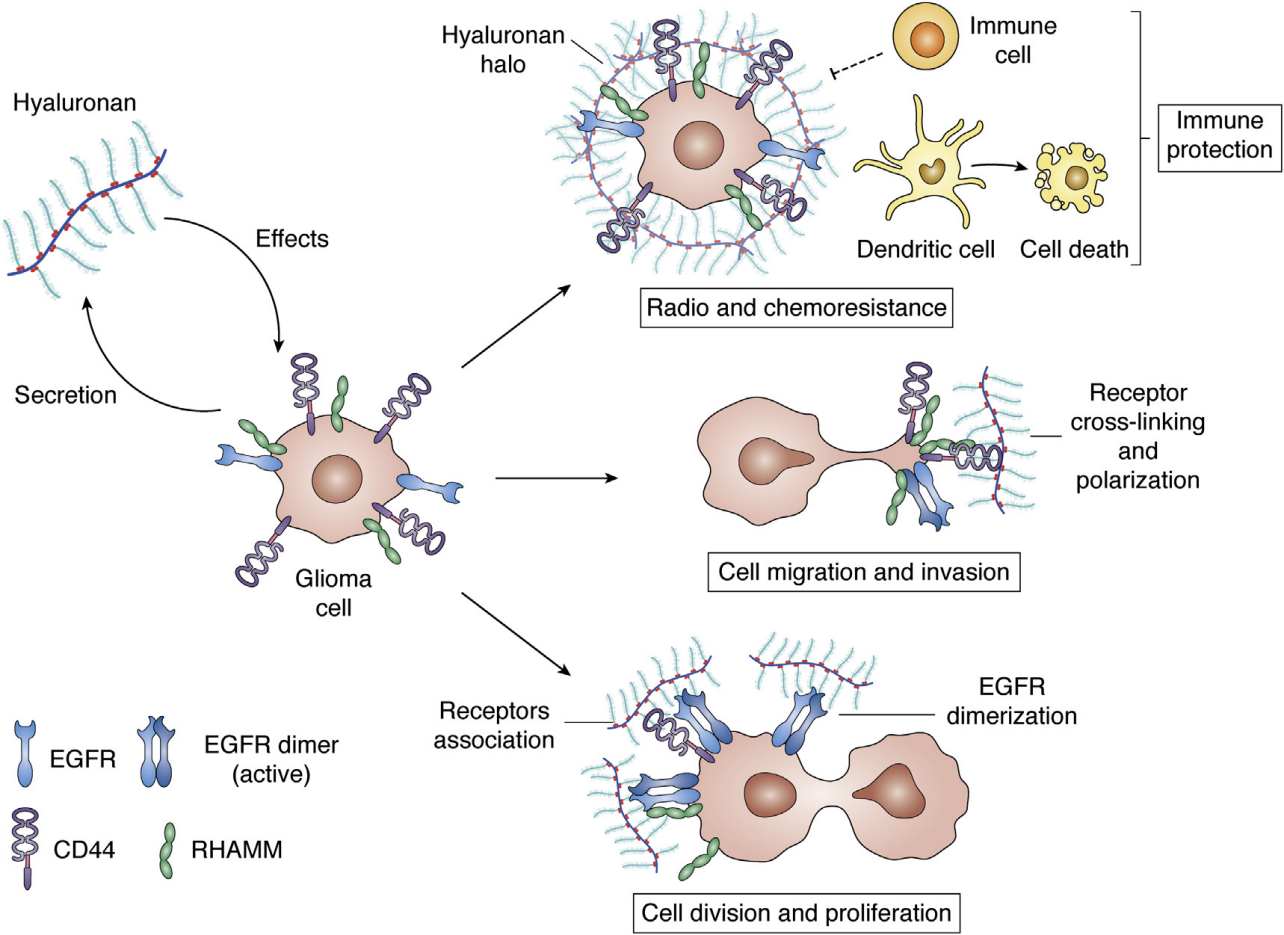
**Impact of HA on GBM biology.** Hyaluronan impacts glioblastoma cells through interaction with its receptors CD44 and RHAMM, which also interact with other receptors, such as EGFR, to increase migration, invasion, proliferation as well as radio and chemotherapy resistance. Furthermore, glioblastoma cells secrete HA, which forms a halo that induces dendritic cell death and hinders the action of immune cells generating an immunoprotective barrier. Therefore, HA enhances GBM progression by amplifying features of malignancy and suppressing immune attack.

In this review, we examine the main questions facing the field, hoping to shed light on this important disease. For example, which of HA’s many roles impact GBM development? How does the 3D structure of HA influence its effects on GBM cells? Does the effect of HA depend on its concentration or quality? Are interactions with CD44 (58) and/or RHAMM (59) important, and what other biomolecules might be involved? Given previous observations of increased HA in the tumor context and its successful use as a prognostic marker in other pathologies, can HA be used as a biomarker for this particular neoplasm? Is HA itself really an appropriate therapeutic target, and if so, how can we best influence HA function?

We first describe existing evidence of HA’s impact on GBM and the receptors and broader signaling pathways that mediate HA functions, including both *in vitro* and *in vivo* evidence. We examine how the 3D structure of HA impacts its effects *versus* soluble HA on GBM cells. We consider the correlations of HA and HA-related molecules with tumor grade and GBM patient survival. Finally, we explore the potential of HA as a therapeutic target, connecting both *in vitro* and *in vivo* approaches, and consider therapeutic alternatives to target HA.

## How hyaluronan and its receptors alter glioblastoma biology

**Figure 2:**
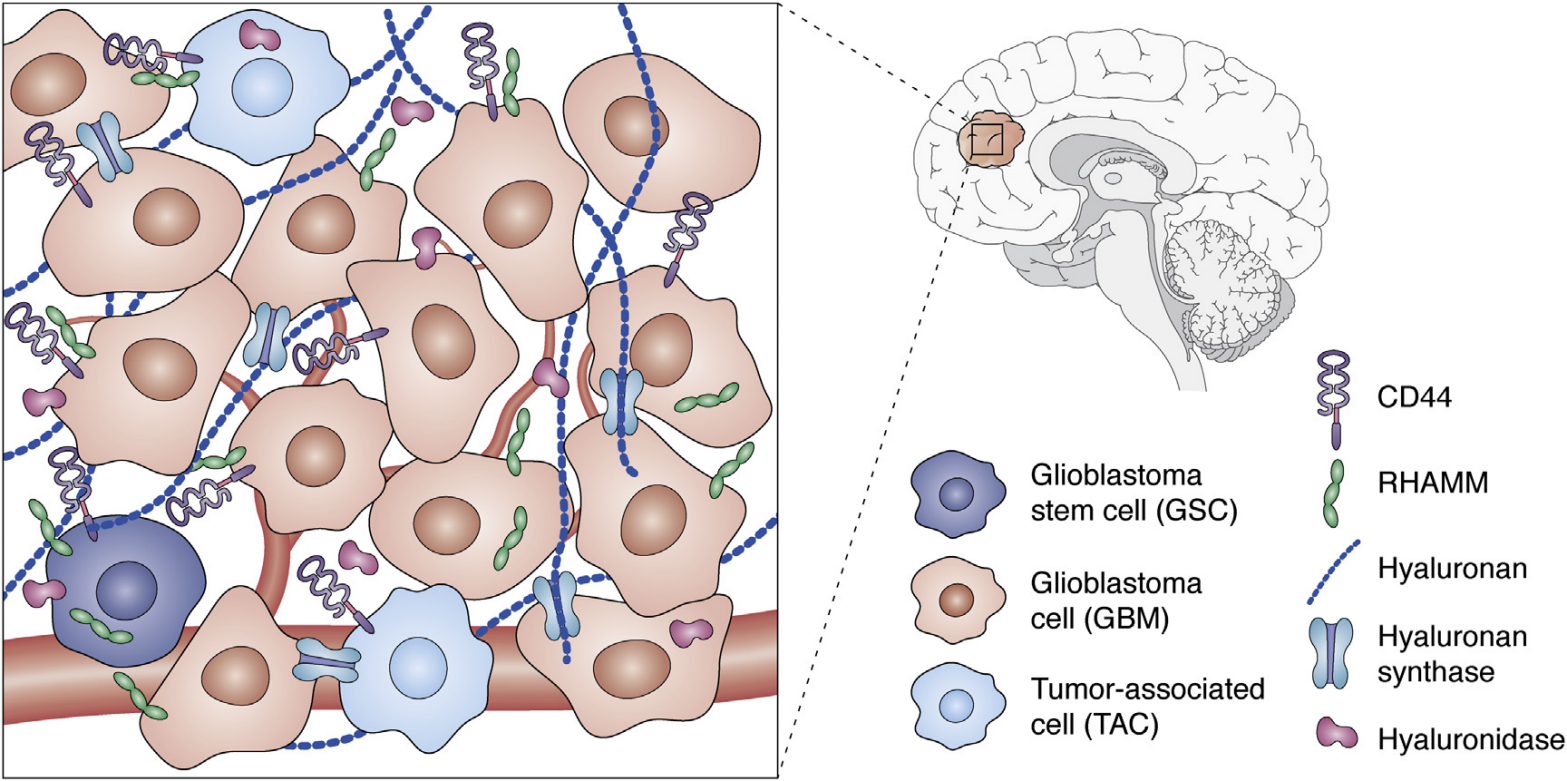
**HA and HA-related molecules in samples of GBM patients.** The GBM microenvironment is a complex scenario in which GBM cells and GSC are intermixed with tumor-associated cells (TAC). In addition, there are multiple matrix components, such as HA being the main GAG in the CNS. The molecules related to its metabolism are also important in GBM invasion. This fact is reflected in the presence of RHAMM and CD44 on the edge of the tumor. It is noteworthy that although HA failed as a molecular marker, the enzymes responsible for its synthesis (especially HAS2) and its degradation (particularly HYAL-2) are associated with tumor grade and even correlate with patient outcomes.

As discussed above, HA plays many physiological and pathophysiological roles. Here we capture HA’s functional impacts on GBM cells and introduce the biomolecules that mediate these effects, including its receptors RHAMM and CD44, coreceptors, and HA-binding proteins (60, 61, 62, 63, 64) (Fig. 2).

CD44 is a multifunctional transmembrane glycoprotein that belongs to the group of link-module proteins and is expressed in numerous cells and tissues (65, 66). There are several CD44 protein variants encoded by alternative splicing involved in various biological processes, such as migration and cellular adhesion, lymphocyte homing, as well as cellular differentiation and proliferation (67). In the CNS, CD44 has been implicated in neuronal development and in the response to injury (68, 69), while mice lacking this receptor have deficits at neurological levels (70). Even though its main ligand is HA, CD44 can interact with other molecules such as collagen, fibronectin, laminin, osteopontin, growth factors, and metalloproteinases (MMPs) (71, 72). Several tumor cells overexpress CD44, and it has been related to tumor progression, apoptosis evasion, and multidrug resistance (49, 52, 73, 74, 75). Indeed, in GBM, CD44 is strongly involved in cell invasion (68). Moreover, it was demonstrated that optimal levels of CD44 were necessary on GBM cells to generate highly infiltrative tumors in a mouse model (76, 77) and that treatment with an anti-CD44 monoclonal antibody inhibited tumor growth of local glioma in a mouse model (78). These reports implicate CD44 in HA-enhanced proliferation and migration.

Beyond cell migration, HA and CD44 were shown to be involved in the ability of GBM cells to evade immune attack. In the CNS, immunity depends on effective innate immune activity, since most requirements for adaptive immune responses are not available (79). Thus, microglia and resident macrophages are the key immune effector cell populations in the brain. Physiologically, the CNS parenchyma is secluded from circulation by the blood–brain barrier (BBB), a specialized endothelial barrier formed by endothelial cells, pericytes and astrocytes, which controls the passage of water to the brain among other functions (79). In the presence of GBM, the BBB is impaired, resulting in an infiltration of monocytes and other immune cells from the periphery (80). The microglia and peripheral macrophages recruited to the glioma environment are known as tumor-associated macrophages (TAMs) (81, 82). Yet, cancer cells can subvert the immune functions of these cells, using excreted factors, such as HA, to re-educate TAMs to facilitate tumor proliferation, survival, and migration (83, 84).

Although dendritic cells (DCs) are less studied than TAMs, DC inoculations are an interesting strategy to attract and stimulate brain-specific T cells (85). However, HA synthesized by 9L glioma cells interacts with CD44 to promote apoptosis of DCs *via* induction of iNOS (86). Moreover, glioma cells grow HA “halos” when in coculture with peripheral blood mononuclear cells (PBMCs). These halos impede the contact between different cell types and reduced the generation of specific T cells for glioma antigens, which may constitute a suppressor mechanism to evade the cellular immune attack (87, 88) (Fig. 1). Interestingly, while treatment with the immune cell-activating interferon-α/β does not modify HA secretion, incubation with anti-inflammatory glucocorticoids decreased HA levels in the glioma culture medium (89, 90), suggesting that an inflammatory context would be necessary for HA production and GBM progression. Finally, recent data demonstrated that tumor-associated mesenchymal stem-like cells secreted C5a, which activates ERK and triggers expression of the HA synthase HAS2, contributing to HA abundance and enhancing GBM invasiveness in a nude mice model (91). These results provide compelling examples of the ways in which tumor cells modulate their microenvironment to favor their own malignant behaviors. Moreover, they show that HA can influence GBM progression by enhancing GBM malignancy, increasing proliferation, migration, and invasion, and by impairing the attack of the immune cells.

CD44 is also associated with the specific binding of glioma cells to HA, but it is not the only receptor involved (92, 93, 94, 95, 96).

RHAMM has several isoforms obtained by alternative splicing (97) and is found in the cytoplasm, nucleus, on the cellular surface, and even in cell culture supernatant (98, 99, 100, 101). It has been proposed that the levels of RHAMM in the cytosol must be carefully balanced to enable the correct formation of the mitotic spindle and for genomic stability (99, 102, 103). Within the CNS, RHAMM has also been associated with glial motility in response to CNS injuries, axon extension, mitochondrial trafficking, and in the folding of the neocortex in fetal human brain (104, 105, 106, 107, 108, 109, 110, 111). In contrast to CD44, RHAMM is normally expressed in only a few tissues. However, RHAMM is considered a tumor-associated antigen, expression of which is increased during malignant cell transformation (98, 112, 113). Particularly in GBM, RHAMM has been associated with an increase in migration and proliferation, and its levels have been correlated with tumor grade (91, 114, 115, 116). For example, in 2001, it was demonstrated that high-grade gliomas expressed higher levels of RHAMM and CD44 than low-grade lesions and that RHAMM inhibition hindered proliferation and migration of glioma cells, both in the presence and in the absence of HA-based ECM (115). It is worth noting that interpreting the effect of RHAMM inhibition in the absence of the HA-based ECM is complicated by the fact that HA can be secreted by GBM cells. Finally, in an *in vivo* xenograft model, it was demonstrated that RHAMM silencing reduced tumor formation and extended mouse survival time, while its overexpression enhanced tumor growth, compared with control (117). These results highlight the relevance of RHAMM, and presumably its ligand HA, in GBM progression.

While these data establish the clear importance of CD44 and RHAMM in mediating HA’s functions, they are not the only biomolecules involved. As mentioned above, GBM is a heterogeneous disease. It has been linked to multiple oncogenic alterations such as mutations in *tp53* and *atrx* (α-thalassemia/mental retardation X-linked syndrome) *pten* (phosphatase and tensin homologue) *tert* (telomerase reverse transcriptase) and *h3f3a* (histone H3.3) genes, codeletion of chromosome arms 1p and 19q, monosomy of chromosome 10, gains of chromosome 7, and *egfr* (epidermal growth factor receptor) and *pdgfra* (platelet-derived growth factor receptor-α) gene amplifications (118, 119, 120). Interestingly, the last two alterations have in turn been linked to changes in ECM composition. For instance, GBM tumors overexpressing PDGFRα showed elevated expression of chondroitin sulfate proteoglycan 4 (CSPG4/NG2), aggrecan, and extracellular sulfatase 2. In contrast, the GBM cases with EGFR amplification were associated with increased CSPG4/NG2 expression, but with diminished aggrecan and extracellular sulfatase 1 (121). Although aggrecan is able to bind HA, the direct relationship between these molecular alterations and HA levels in the ECM remains unknown and could be an interesting field to explore for GBM classification and treatment in the future.

EGFR has been studied in the context of GBM separately (122), but its association with the HA receptors could explain some of the effects of HA on GBM progression. For example, it was demonstrated that CD44 binds EGFR, and it was postulated that the complex may provide a mechanism for HA-mediated cell invasion and proliferation (123). RHAMM is also known to associate with transmembrane receptors including CD44, CD44/EGFR, PDGFR, TGFβR-1, bFGFR, and RON (100, 124, 125, 126, 127, 128). In this way, RHAMM can modulate the pathway associated with each one of these receptors and control the expression of genes involved in the cell cycle, influencing proliferation and cellular migration (98, 103, 112, 124, 129). Therefore, it seems reasonable to think of these complexes as signaling transducers. In addition to these membrane interactions, CD44 has been shown to partner with the intracellular moesin, a protein that connects the actin cytoskeleton to the plasma membrane. This interaction occurs after HA treatment, enhancing U87MG and U373MG glioma cell migration and invasion (130).

Several other HA-binding proteins have also been identified. Jaworski *et al.* (131) reported that the extracellular hyaluronan-binding protein known as brevican or brain-enriched HA binding (BEHAN) is consistently expressed by human gliomas and enhanced HA-mediated glioma invasion, especially when cleaved by disintegrin and metalloproteinase with thrombospondin motifs 4 (ADAMTS4) (132, 133). Finally, it was demonstrated that HA- and proteoglycan-link protein 4 (HAPLN4) increased adhesion and migration and even potentiated the motogenic effect of brevican on U251MG and U87MG cells (134).

These combined results paint a picture of growing complexity, in which assigning specific GBM-promoting functions becomes scrambled by the multiple receptors, coreceptors, complexes, and regulatory factors potentially involved. However, HA emerges from this scrambled scenario as the key player that brings together the variety of effects observed in GBM cells, emphasizing the potential of HA as a target for improving GBM treatment. In addition, CD44 and RHAMM rise as the main players to watch over HA actions in the context of GBM. But how do CD44 and RHAMM perform this role? We next explore the downstream signaling pathways that execute HA-mediated functions.

## Signaling pathways involved in HA effects

**Figure 3:**
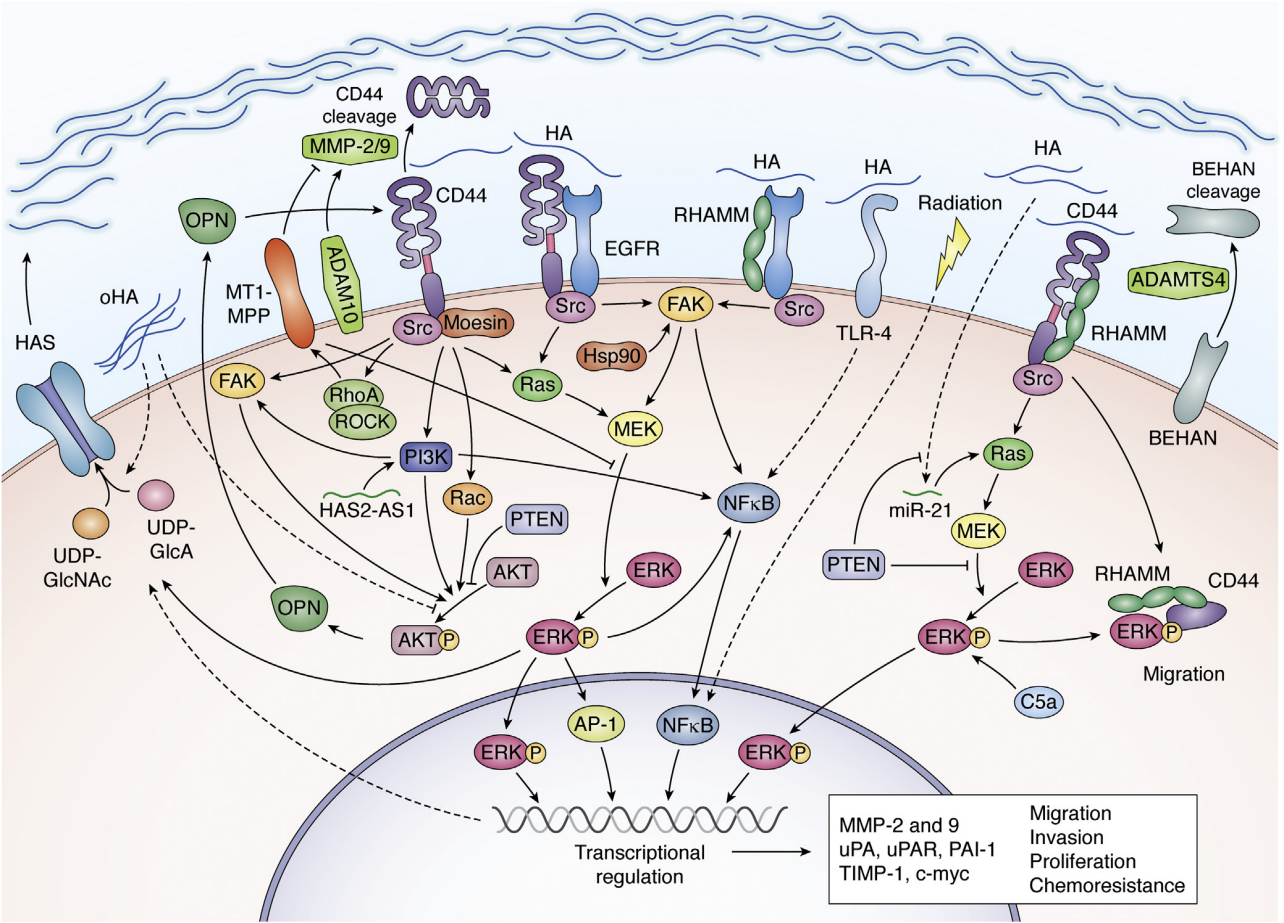
**The scrambled relationship between GBM and HA.** HA is accumulated in the GBM microenvironment. Additionally, GBM cells overexpress its receptors, CD44 and RHAMM, as well as tyrosine kinase receptors such as EGFR. In this context, the interaction of HA with CD44, RHAMM, probably in a complex with EGFR, and even TLR4 leads to an hyperactivation of signaling pathways such as MEK/ERK and PI3K/Akt and the overexpression of transcription factors, mainly NFkB. The final molecules of these signaling pathways along with NFkB impact on a transcriptional regulation enhancing all the malignant features of GBM, mostly proliferation, migration, and invasion but also chemoresistance. Moreover, the activation of these signaling pathways increases the activity of MMPs, which cleave CD44 and increase GBM migration and invasion. The tumor suppressor gene, PTEN, inhibits several of these pathways; however, almost 40% of GBM are PTEN-negative. Finally, the transcriptional regulation and, unfortunately, the radiation therapy enhance HAS activity, increasing the HA amount in the GBM microenvironment, leading to a positive feedback loop and favoring GBM progression. The complexity of this scrambled story hinders the understanding of HA effects on GBM cells, at the same time highlighting the potential of HA as a therapeutic target to improve the outcome of GBM patients.

As discussed above, HA interacts with several receptors and coreceptors, potentially triggering a multitude of intracellular pathways (Fig. 3). For example, using the U87MG and SMA560 glioma cell lines, Tsatas *et al.* showed that treatment with HA (70 μg/ml) resulted in an increase in ERK1/2 phosphorylation in an EGFR-dependent manner. Conversely, the disruption of the CD44/EGFR complex reduces ERK1/2 activation in U87MG glioma cells (135). The EGFR receptor is involved in the hyaluronan-induced expression of urokinase-type plasminogen activator (uPA), urokinase-type plasminogen activator receptor (uPAR), plasminogen activator inhibitor-1 (PAI-1), tissue inhibitor of metalloproteinases (TIMP-1), and c-myc pathway (123). All these molecules and signaling pathways were associated with an enhanced proliferation and migration in GBM.

PTEN is a tumor-suppressor gene whose loss is extremely frequent in GBM (40%), which is correlated with enhanced proliferation as well as TMZ resistance (136). This correlation was explored in a study that expressed PTEN in U87MG cells lacking functional PTEN (137). Treatment of these cells with 100 μg/ml HA significantly decreased the levels of MMP-9 and MMP-2, inhibiting the activation of ERK1/2 and focal adhesion kinase (FAK) while increasing the levels of tissue inhibitor of MMP-1 and MMP-2, which resulted in the inhibition of cell invasion (137). In a second study, 200 μg/ml HA induced the expression of osteopontin, a protein related to GBM migration and invasion, through activation of the PI3K/Akt pathway in a PTEN-dependent manner (138). Finally, PTEN suppressed HA-induced miR-21 expression, an interference mRNA that increases the strength and duration of Ras/MAPK signaling, enhancing MMP-9 expression and glioma invasion by downregulation of Spry2 (139). Overall, the loss of PTEN seems to create an opportunity for the HA-stimulated ERK1/2 and PI3K/Akt signaling pathways to promote the levels of MMPs, which in turn promotes invasion and migration.

Two studies converged on these same pathways from alternative starting points: Kim *et al.* (140) demonstrated that Emodin, an inhibitor of tyrosine kinase proteins, significantly inhibited HA-induced invasion and the secretion of MMP-2 and MMP-9 in U87MG cells *via* inhibition of FAK, ERK1/2, and Akt activation, as well as partial inhibition of two transcription factors, activator protein-1 (AP-1) and nuclear factor-κB (NF-κB). The same group showed that the inhibition of Hsp90 reduced HA-induced migration and invasion as well as MMP-9 secretion through FAK inhibition, which impedes NF-κB activation (141). These studies thus add the FAK signaling pathway and transcription factor NF-κB as components relevant in the migration, invasion, and proliferation of GBM cells.

Across all of these reports, MMPs are the common target where the signaling pathways converge. However, MMP-2 and MMP-9 are collagenases, and the brain ECM is mostly composed of HA, making it unclear how their enzymatic activity would effectively create openings in the matrix to facilitate migration/invasion. While this activity may be critical for neoangiogenesis (142, 143), the link to migration seems to be through an indirect route: Chetty *et al.* (144) showed that MMP-9 induced cleavage of the extracellular domain of CD44 in xenograft glioma cell lines, promoting cell migration. Similarly, MT1-MMP, a membrane matrix collagenase, mediated CD44 cell surface cleavage through MEK/ERK and RhoA/ROK pathways, inducing detachment of the cell from the HA substrate and promoting the invasive potential of glioma cells (145, 146).

Adding more complexity, MMPs are not the only triggers of CD44 cleavage. In fact, CD44 can promote its own cleavage: CD44 activation stimulates Rac-mediated cytoskeletal rearrangements that increase CD44 cleavage by disintegrin and metalloproteinase 10 (ADAM10), potentially contributing to the migration and invasion of U251MG cells (147). Modifications to membrane composition through short-term cholesterol depletion similarly augmented CD44 shedding mediated by ADAM10 and caused changes in CD44 localization, while long-term cholesterol reduction suppressed the CD44 cleavage-induced cell migration on a hyaluronan-coated substrate (148). Interestingly, Lamontagne and Grandbois demonstrated that unstimulated U373MG cells interact with HA through CD44 receptors across their entire surface, while in protein kinase C-activated cells, the interactions were localized at the leading edge of the cells (149). These results suggest that not only changes in CD44 expression levels but also even its localization is important in the process of glioma cell migration.

These studies collectively demonstrate a role for CD44 in HA signaling. RHAMM has been less studied than CD44, but we suspect that this receptor and downstream signaling pathways are similarly important for HA-mediated GBM progression and would benefit from further investigation. Finally, we must consider the nature of the ligand that binds to these receptors. How does the 3D structure of HA, which remains not fully clarified, determine its function?

## Mimicking the brain: How a complex environment impacts GBM biology

As discussed above, extracellular HA forms a functional scaffold for other ECM components, which can be extended *via* extrusion of additional HA by hyaluronic acid synthases to form protective halos or other structures. The molecular mass of the HA polymer affects the number of cross-links that can be formed and the HA saccharides available for receptor binding and thus is a critical factor in its actions, as shown on a GBM cell line (150, 151). For example, in a cancer context, HMW-HA accumulation is frequently associated with cancer progression, whereas LMW-HA promotes the development of the tumor and o-HA attenuates its development. These disparities are explained by the different manners of interaction with the HA receptors, CD44 and RHAMM (152). Unfortunately, most HA research usually fails to report such data, which hinders the general conclusions that can be drawn about HA’s effects. Moreover, exploring these different molecular sizes, while of value, does not necessarily provide insights into the true tumor microenvironment. Actually, the fact that HA is organized into a 3D structure in the brain ECM suggests a complex scenario that may be difficult to reproduce *in vitro* by the exogenous addition of soluble HA limiting our ability to fully interrogate HA-mediated mechanisms. Data using HA hydrogels have provided an important entry into this area. Indeed, 3D culture systems have been used to reproduce cancer cell behavior more faithfully than common 2D cultures of various malignancies including GBM (153, 154, 155). Their use in preclinical assays has been proposed as they show better conservation of real growth and sensitivity to drugs and radiation than 2D cultures (156, 157, 158, 159).

The first report using HA hydrogel to mimic the brain ECM was published in 1997 (160) and was followed by several studies, which centered their attention on the differences in the stiffness and the elastic modulus of the hydrogel rather than on the effect of HA *per se* (161, 162, 163, 164, 165, 166, 167, 168, 169, 170, 171, 172). In the first studies in which HA was used to stimulate glioma cell migration in a complex 3D approach, the authors reported that the effect might be indirect and partially due to gel polymerization and network structure (59, 173, 174). These combined studies demonstrated the importance of considering the maintenance of the elastic modulus, porosity (162, 165, 166, 167, 168, 169, 170, 173), and composition of HA hydrogels as compared with HA-free hydrogels when assessing cell features in complex environments (175). Comparison of several recent studies provides an excellent demonstration of these aspects. First, two studies showed that GBM cell migration would be inversely proportional to HA; however, in one of them, the elastic modulus and porosity of HA hydrogels varied with the amount of HA added to the hydrogel (176), while in the other study, N,N-dimethylformamide was used in the HA hydrogel but not in the other scaffolds, potentially hindering clear conclusions (177, 178). Other reports that controlled for stiffness and porosity of the gels with and without HA showed that HMW-HA increased migration and haptotaxis of glioma cells (179, 180, 181). Reinforcing these findings, it was demonstrated that the addition of HMW-HA to hydrogels increased the proliferation and migration of patient-derived GBM stem cells (GSCs) and the expression of epithelial-to-mesenchymal transition (EMT)-related genes on U87MG GBM cells, enhancing their *in vivo* tumorigenic ability, with respect to cells cultured in scaffolds without HA (182, 183). In accordance, another work reported that U87MG cells in the presence of 3D hydrogels containing 1500 to 1800 kDa HA showed characteristics resembling the stem cell phenotype, including an increased expression of the markers RHAMM and CD133, compared with hydrogels without HA (184).

With regard to the importance of the molecular mass of HA in its effect, it was shown that patient-derived xenograft cells cultured in hydrogels containing 500 kDa HA showed less invasiveness than those in hydrogels containing 10 or 60 kDa HA (151). However, a separate study demonstrated that the addition of HA (60 kDa) to the hydrogel did not affect metabolic activity but reduced invasiveness in U251MG cells (185). Overall, these data suggest that, when the stiffness and porosity of 3D structures are considered, the effect of HA is similar to that seen in assays using soluble HA, enhancing GBM proliferation and migration. However, the response of GBM cells depends not only on the model but also on HA concentration and molecular mass.

HA hydrogel stiffness also impacts adhesion and migration speed, implying that CD44 signaling is mechanosensitive (186) and pointing to additional biomolecules involved in mediating HA activity: CD44 suppression in U373MG and U87MG human GBM cells reduces cell adhesion to HA at short times (0.5 h) while maximal adhesion at 3 h requires both CD44 and integrins. In another study using U87MG cells, the presence of 1630 kDa HA in gelatin or polyethyleneglycol gels also caused cluster growth and modified the expression of fibronectin, MMP-2, MMP-9, vascular endothelial growth factor (VEGF), and hypoxia inducible factor 1 (HIF-1) depending on EGFR status and HA concentration (187). A second report echoed these conclusions, finding that matrix-bound HA enhances metabolic activity and proliferation depending on the EGFR status of U87MG cells and favors invasion under hypoxia (188). Though both VEGF and HIF-1 were already known to be involved in the malignancy of GBM, these reports help to clarify a possible mechanism for their role, connecting HA to EGFR, probably in a complex with CD44 or RHAMM, and thus to an increase in HIF-1 and VEGF levels.

Using 3D conditions has also revealed new details regarding the role of HA in drug resistance. A HA/CD44/PI3K/Akt axis has previously been proposed as a pathway involved in drug resistance in several types of tumors (189, 190, 191, 192). Recent evidence has extended this mechanism to GBM. Specifically, it was shown that the addition of 1630 kDa HA to hydrogels boosted metabolic activity of patient-derived xenograft cells (193). In the same work, it was shown that HA upregulated genes associated with matrix remodeling and tumor growth generating resistance to the EGFR inhibitor erlotinib depending on CD44/PI3K axis. However, in cells expressing EGFR^vIII^, a constitutively activated mutant of EGFR, these effects remain unaltered despite CD44 blocking, thus suggesting the implication of other pathways activated in EGFR^vIII^ within HA matrices (193). Besides, in a HA hydrogel, CD44 knockdown restored the cytostatic and cytotoxic effects of erlotinib on resistant GBM cells. However, GBM cells recovered resistance after 12 days. Under such conditions, the authors reported an RHAMM expression pattern resembling that of CD44, which would be able to compensate the resistance in the absence of CD44 (194). In fact, RHAMM has been implicated in drug resistance in other tumors, through activation of signaling pathways such as PI3K/Akt and TGFb/Smad2 (49, 195, 196). Thus, its study as well as the pathways that trigger would expand our understanding of HA-mediated drug resistance and would be an interesting starting point for future investigations on GBM therapy.

Overall, the effects of HA observed in 3D assays are certainly similar to those reported using 2D culture approaches in which soluble HA is added to the culture medium, including the same receptors and signaling pathways. However, invasive features are dependent on the composition, elastic modulus, and porosity of hydrogels, and groups that have not considered these variables have reported controversial HA effects regarding those features. Moreover, 3D cultures enable evaluations of additional aspects of GBM biology, such as the mechanosensitive CD44-driven effects. Future research will benefit from additional reporting of hydrogel preparations, ingredients, and material properties, and for conclusions drawn to carefully consider whether outcomes can be linked to the effect of one component of the hydrogels. Increasing incorporation of advanced models for GBM will bring us closer to understanding the complicated details of tumor development, which should similarly increase our ability to diagnose and treat this disease.

## Hyaluronan and its metabolism as a molecular marker for glioblastoma

Given the effects of HA on GBM biology, an obvious question is whether HA could be used as a biomarker for this cancer type. Indeed, it was revealed that the quantity of GAGs in malignant tissue samples, particularly in GBM, was considerably higher than that of nontumor specimens (197). In addition, Delpech *et al.* (198) showed that in glial tumors the HA content was much higher than in adult normal brains, mainly due to increased quantities of HMW-HA. HA content was also higher in GBM tissue than in lung carcinoma metastases (199). However, the proportion of HA in comparison to other GAGs was lower in GBM than in other low-grade gliomas (197), and it was even shown that the concentration of mucopolysaccharide acid, particularly HA, was inversely associated with glioma grade (200, 201). It is worth noting that the molecular mass of HA might be underestimated in these reports, which would be an important consideration for further analysis. For now, however, it seems unlikely that HA concentrations could be used for diagnosis. Could its localization point to clusters of GBM cells?

HA is found both within and surrounding the tumor in an intracranial mouse model (194). Although the GBM parenchyma was slightly stained for GAGs in human samples, it was observed that the infiltrated cortex areas exhibited strong staining for HA and CS, suggesting a heterogeneous distribution (202). Likewise, it was demonstrated that HA was localized in pericellular and perivascular areas, suggesting that both cancer cells and tumor-associated vascular cells secrete it (198). It would be interesting to determine plasma levels of HA and their relation, if any, with tumor stage. However, the current data suggest that an alternative molecule is needed.

The observed rise in HA levels in brain tumors was accompanied by variations in the content and distribution of other HA metabolism-associated proteins. For example, both HA and RHAMM were found to be elevated in the tumor parenchyma, particularly in the invasive front, suggesting their crucial role in GBM invasiveness (115). Considering that both RHAMM and CD44 are involved in HA-mediated invasion in GBM, it would be expected these receptors to be highly expressed in the migratory edge. In agreement with this, it was reported that CD44 expression was augmented in the glioma–brain interface of a G26 glioma-bearing mouse model, with single infiltrating CD44-enriched cells escaping into the brain parenchyma (203) (Fig. 2). Strangely, although both HA and CD44 levels were increased in human and rat GBM samples, they showed an opposite distribution, with CD44-enriched regions overlapping with lower HA content (204). This discrepancy might make it difficult to interpret diagnostic readings.

Another obvious set of candidates are the enzymes responsible for the synthesis and degradation of HA, as these are primarily responsible for controlling HA levels (205, 206, 207). HA is synthesized by hyaluronic acid synthases (HASs), which extrude the polymer to the extracellular environment as is produced. Three isoforms of these proteins have been reported, HAS1–3, each with distinct functions (208, 209). HA degradation, where the most stringent regulation occurs, is performed by the hyaluronidases (HYALs). There are six HYAL-like genes in the human genome: hyal-1–4, ph-20, and the pseudogene pHyal1 (210, 211). Similar to the HASs, each HYAL has differential effects on HA (208, 210, 211, 212, 213). Several studies have examined the possible correlation of HASs or HYALs with GBM prognosis. For example, a connection has been observed between the expression of HAS2 and GBM (199). Valkonen *et al.* recently extended these results, reporting tissue microarray data showing that HA and HAS1-3, the HYALs, and CD44 were highly expressed not only in malignant tissue, but also in adjacent gliotic cerebral tissue of grades II to IV diffusely infiltrating astrocytomas. In this study, although the HA content did not exhibit a prognostic value, an association between tumor grade and HAS1, HAS2, and HYAL-2 was observed, and an increase in HAS2 was also correlated with a poor prognosis of the patients (214). Unexpectedly, HAS2 overexpression in GBM cells abolished tumor formation in the brain in a mouse model; the authors proposed that this was due to a lack of HYAL activity, suggesting that gliomas may be more aggressive when coexpressing HAS and HYALs (215).

These data suggest that HA metabolism seems to be crucial in GBM, and HA degradation could be more relevant than HA synthesis. However, it’s worth noting that HA synthesis consumes energy and so is regulated by metabolic sensors such as UDP-GlcNAc, AMP-activated protein kinase (AMPK), and sirtuin 1 (SIRT1) (216). HA synthesis is also regulated at the epigenetic level by HAS2-AS1, an HAS2 antisense transcript (217, 218, 219). Whether any of these molecules might have potential as GBM biomarkers has not been explored.

## Hyaluronan as a therapeutic target in glioblastoma

The increases of HA in tumor tissues with respect to the healthy brain discussed above may not have obvious diagnostic potential, but they do implicate HA in GBM progression and suggest its potential as a therapeutic target. New targets are desperately needed for GBM, as the median survival time after diagnosis is only 14.6 months. Moreover, existing treatments can impact healthy brain tissue in addition to disrupting GBM progression. For example, radiotherapy induces a disruption in the BBB, which would enhance the effect of chemotherapy on GBM cells but also expose normal cells to the same chemicals or other complications due to loss of BBB integrity (220). Moreover, treatment with TMZ seems to activate the immune system against the tumor cells (81, 82). However, the drug could also generate DNA damage on normal cells that can have negative consequences on the function of the normal brain, diminishing the quality of life of patients.

HA degradation has been explored as a therapeutic target in several studies (68, 76, 77, 117, 221, 222). Clinical trials have shown that treatment with HYAL as adjuvant chemotherapy improves drug biodistribution and particularly access to the tumor site, contributing to clinically relevant remissions in high-grade astrocytomas and to the efficacy of chemotherapy in pediatric brain tumors (223, 224, 225). In addition, it was recently reported that treatment with HYAL, alone or in combination with TMZ, showed a cytotoxic effect on the GSC population. Furthermore, HYAL treatment results in upregulation of CD44 and a decrease in stem cell phenotype (226). In agreement with this, it was reported that a reduction of CD44 expression increased GSC features (204). These findings suggest that targeting HA can have direct effects on CD44 and the GBM stem cell phenotype.

Interestingly, it was observed that treatment with HYAL increases HA synthesis (222, 227, 228), which could be due to a compensatory mechanism. Moreover, one report did show an increase in U87MG cell proliferation after HYAL treatment (229). Thus, although most of the consulted bibliography reported antitumor results, it is clear that our understanding of HYAL in GBM treatment is incomplete, and new ideas regarding the role of HYAL are needed.

The *in vitro* treatment of HA with HYAL generates oHA. Therefore, we propose that the effect of HYAL treatment on glioma cells could be partially explained by the generation of oHA in the medium. These oHA occupy the HA-binding site of the receptor and, because of their small size, fail to cross-link receptors, preventing signaling transduction and the normal consequences of native HA. This proposal is supported by data showing that the treatment with oHA decreased proliferation, downregulated both Akt activation and expression of breast cancer resistance protein (a transporter associated with drug resistance in cancer progenitor cells), and caused increased apoptosis in C6 cells (230). Reinforcing the opposite effects between oHA and native HA, it was proposed that the last increases the levels of ceruloplasmin, a protein related to hypoxia, inflammation, and angiogenesis in gliomas, while oHA causes a reduction in its levels (231).

Prior work has explored the direct application of oHA. It improved the effects of both radiation and methotrexate treatment on U87MG cells and enhanced the effects of TMZ, carmustine, and MTX in C6 rat glioma cells (230, 232, 233). Related work has shown that radiation leads to an aggressive phenotype. For example, Yoo *et al.* (234) showed that radiation treatment enriches the HA content in the tumor microenvironment through NF-κB activation, enhancing the invasive properties of GBM cells and leading to poor prognosis. Similarly, it was shown that subcurative radiation enhanced cell proliferation and increased MMP2 and CD44 expression beyond the tumor periphery (235). Taking into account that HA interacts with Toll-like receptor 4 (TLR4) and activates NF-κB, which leads to enhanced proliferation in GSC (236), the finding of Yoo *et al.* could explain the fact that oHA treatment improves the radiotherapy effect on GBM cells, since it might inhibit the protumor effect of HA induced by radiation.

An alternative strategy is to block synthesis of HA directly. We recently studied the effects of the inhibitor, 4MU, which is known to inhibit HA synthesis but seems to also act *via* HA-independent mechanisms that are just beginning to be understood. We demonstrated that 4MU decreases metabolic activity, cell proliferation, migration, and MMP-2 activity while causing high levels of apoptosis in murine GL26 GBM cells. Moreover, we showed that 4MU partially diminishes HA synthesis, although several of its effects would be independent of this inhibition (46).

Another direction being explored is the use of HA to deliver other drugs and reagents to GBM cells, taking advantage of their overexpressed HA receptors (237). For example, in 2015, Cohen *et al.* developed HA-coated lipid-based nanoparticles (HA-LNPs) that could deliver PLK1 siRNA. They used BALB/c nude mice inoculated with U87MG cells and observed that only those LNPs treated with HA showed specific binding to glioma cells that increased over time, which was attributed to an HA–CD44 interaction (238). Similarly, Liu *et al.* developed a novel HA-decorated micelle loaded with gemcitabine and honokiol as chemotherapeutic agents. They showed that this system could effectively cross the BBB and target tumor cells, showing effective suppression of cell proliferation, enhanced apoptosis, and extensive necrosis (239). Another group reported that pairing doxorubicin with HA in a liposomal nanoparticle efficiently targets not only glioma cells, but also cancer stem cells and brain macrophages. In addition, the combination lowered the toxic effects of the drug on the heart and bone marrow and increased mice survival, altogether enhancing its antiglioma efficacy (240). In summary, the use of oHA or HYAL, especially as adjuvant chemotherapy, appear to be promising strategies. Furthermore, in the field of nanotechnology, the use of HA in decorated micelles to target GBM cells is in a growing phase. We also recommend further investigations of 4MU, both to explore its antitumor effects and to learn more about the underlying mechanism of action.

## Conclusion

**Figure 4:**
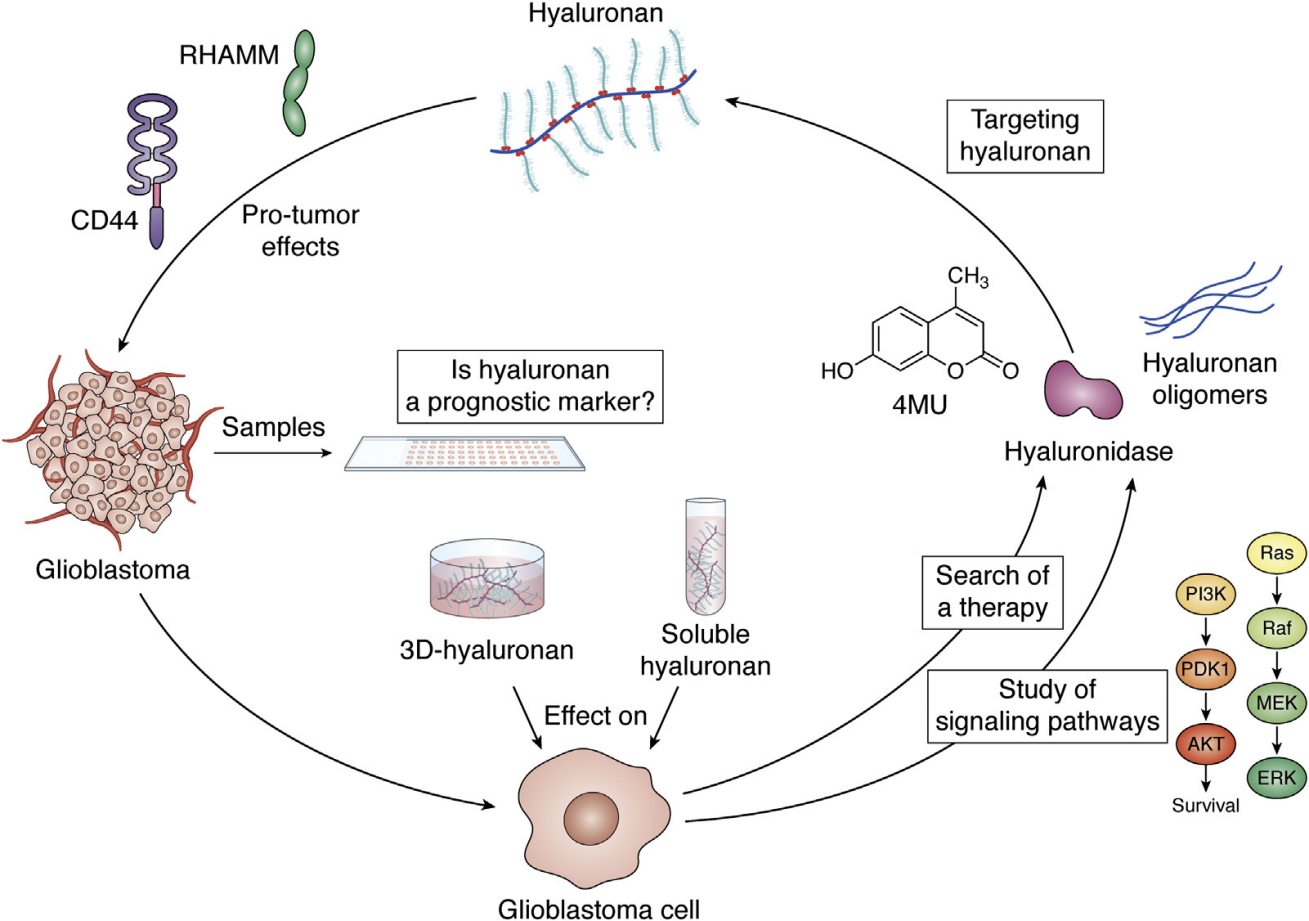
**Overview.** In this review we analyze the effect of HA on GBM biology, highlighting the receptors and signaling pathways involved in these effects. Likewise, we describe differences and similarities observed in the behavior of GBM cells when treated with HA in its soluble form and as part of a 3D structure. We analyze the possible use of HA as a molecular marker for GBM disease. Finally, and taking together all these topics, we attempt to answer the questions regarding the potential of HA as a therapeutic target, discussing and proposing treatment alternatives targeting HA for improving the outcome in GBM patients.

This review seeks to highlight the relevance of HA in GBM progression (Fig. 4). Considering that HA is the main component of parenchymal ECM in the brain, it is necessary to understand its relationship with GBM cells, since the modulation of this interaction might lead to a better patient outcome. As discussed previously, HA is either associated with or related to several proteins implicated in GBM malignancy through several and different interactions and signaling pathways. At the simplest level, HA interacts with RHAMM and CD44, sometimes in complex with EGFR, to activating PI3K/Akt and ERK1/2 pathways, enhancing two key features of GBM malignancy: cell proliferation and migration. However, the plethora of additional mechanisms described and the activation of genes downstream of HA make it difficult to understand its actions. In addition, the different presentations of this GAG (such as HMW, LMW, or oHA) and the various concentrations used for its evaluation have further scrambled the panorama. However, recent exploration of treatments targeting HA, such as the use of oHA, HYALs or 4MU, has shown effective inhibition of the malignant processes in GBM models, providing exciting new directions for GBM therapy. It is imperative to continue with the investigations to fully understand the interaction of HA with GBM cells and, more importantly, to develop an effective treatment that could mitigate its effects to improve survival and life quality in patients with GBM.

## Conflict of interest

The authors declare that they have no conflicts of interest with the contents of this article.
